# Influenza-Related Parotitis during a Large Mumps Outbreak — Arkansas, 2016–2017

**Published:** 2019-05-01

**Authors:** Sarah M. Labuda, Cheng Yang, Carol Daniels, Susan R. Young, Haytham Safi, Dirk Haselow

**Affiliations:** 1 Epidemic Intelligence Service, Centers for Disease Control and Prevention, Atlanta, USA; 2 Arkansas Department of Health, Little Rock, USA

**Keywords:** Influenza, Mumps, Parotitis, Public Health, Influenza-Related Parotitis

## Abstract

During August 2016-July 2017, Arkansas experienced a large mumps (parotitis) outbreak; however, mumps-negative cases of parotitis were also identified in this period. Nineteen of 215 samples (9%) randomly selected for influenza PCR testing were positive for influenza A virus. Practitioners should consider influenza as a cause of nonmumps parotitis.

## Introduction

Mumps virus is the most common cause of epidemic parotitis [1]; however, multiple other respiratory viruses and herpes viruses have been identified in cases of sporadic parotitis [2–6]. During August 2016–July 2017, Arkansas experienced the second largest mumps virus outbreak in the United States in the last 30 years. Among cases of parotitis in the outbreak period, 2954 confirmed and probable cases of mumps virus infection were identified, primarily among Washington County’s Pacific Islander population from the Republic of the Marshall Islands. At the height of the outbreak, standard interviews were not completed in highly impacted settings when >1 case were located in the same address since these cases would by definition be epidemiologically linked. Among the 2954 confirmed and probable cases, 232 were mumps real-time reverse transcriptase polymerase chain reaction (RT-PCR)-negative with no epidemiologic links to other mumps cases. Emergence of mumps-negative cases coincided with local circulation of influenza virus.

Since 1977, influenza A has been identified as a cause of sporadic parotitis [7], and during 2014–2015, it was the most commonly reported virus isolated in a mumps-negative parotitis investigation in the United States [8]. Additionally, influenza A has been identified in sporadic parotitis cases in Spain, Canada, and the United Kingdom [2,5,9,10]. The H3N2 subtype is typically identified when influenza viruses isolated from patients with parotitis are tested further [5,8–10].

On March 2, 2017,the Arkansas Public Health Laboratory (APHL) tested a convenience sample of 8 mumps-negative parotitis buccal swabs, collected during active surveillance of the population in Arkansas for parotitis because of the ongoing outbreak, for influenza and 4 were positive. With 4/8 (50%) of the samples being positive, further examination was planned for the available buccal swabs. The purpose of this study was to determine the extent to which influenza was related to cases of nonmumps parotitis during the mumps outbreak in Arkansas during 2016–2017.

## Methods

During September–November 2017 we examined samples from 3145 buccal swabs that were collected during the period of active surveillance for mumps parotitis (August 2016–July 2017) in Arkansas; the samples were stored in the APHL following the conclusion of the outbreak. A total of 1132 buccal swabs from patients meeting the clinical component of the Council of State and Territorial Epidemiologists standardized mumps case definition of acute parotitis or salivary gland swelling for at least 2 days, but with negative mumps RT-PCR results, were identified. Buccal swabs were stored at APHL at −80C from the time of collection. With a goal of testing 20 samples per month during the outbreak period, we randomly selected 11–20 buccal swabs/month for testing with the CDC Human Influenza Virus Real-Time RT-PCR Diagnostic Panel for influenza A and B using <38.00 cycles as the threshold for positivity. This test is not FDA approved for use or validated on buccal swabs, so was used for study purposes only. First-tier randomization prioritized cases of clinical parotitis with negative mumps RT-PCR results and without epidemiologic linkage to a confirmed mumps case; if fewer than 20 such samples were available in a month, we broadened our criteria to include a 2^nd^-tier of randomized cases of clinical parotitis with negative mumps RT-PCR results with epidemiologic linkage to a confirmed mumps case. Only eight of these cases with epidemiologic linkage to a confirmed mumps case were included in the analysis. To assess mumps risk factors, demographic information for each case of parotitis was collected during the investigation and analyzed descriptively. Chi-square and Wilcoxon rank sum tests were performed in R version 3.5.1. Patients with parotitis in a household with a confirmed case did not have complete interviews at the height of the outbreak due to limited ADH resources and the rapid increase in the number of cases. The Centers for Disease Control and Prevention reviewed this study for human subjects protection and determined it to be nonresearch. Additionally, the Institutional Review Board of the University of Arkansas for Medical Sciences determined this was a public health surveillance investigation.

## Results

Of the 218 randomly selected swabs, 215 were available and tested for influenza A and B viruses. Due to language limitations and other complications encountered in the field, not all interview details were completed. Among 186 swabs for which data were completed on interview forms during the case investigations, the mean number of days from symptom onset to mumps RT-PCR testing was 2.8 +/− 3.3 days, and the median was 2 days (range, 0–21 days). Nineteen swabs (9%) were positive for influenza A virus by PCR, and none were positive for influenza B. Among those positive for influenza, the mean time between onset of symptoms and mumps RT-PCR testing was 2 +/− 2 days, and the median was 1 day (range, 0–7 days). Demographic characteristics comparisons of influenza-related parotitis and mumps parotitis cases are presented in the Table 1. Influenza-related parotitis cases were majority male, white, and spread throughout the state, while mumps parotitis cases were equally split between sexes, majority Pacific Islander/Native Hawaiian, and concentrated geographically in Washington County, Arkansas. Influenza-related parotitis identification in this study mirrored the statewide identification of influenza cases reported during usual seasonal surveillance in Arkansas 2016–2017, with the majority of cases identified in January-March peaking in February. One sample was positive in November 2016; in January 2017, 3 were positive; 7 in February 2017; in March 2017, 6; in April 2017, 1; and in July 2017, 1 was positive. No data regarding influenza vaccine status was available.

## Discussion

This study has findings consistent with other published reports of influenza-related parotitis; cases of influenza-related parotitis were more common among males, compared with the equal sex distribution in cases of mumps parotitis [8]. Additionally, all cases were influenza A, as repeatedly documented as the more common influenza type associated with parotitis [2,5,8–10]. Influenza parotitis in Arkansas was also more common in whites, whereas mumps occurred primarily among the Marshallese population in Washington County. Influenza-related parotitis also occurred among a slightly younger age group than mumps cases.

One strength of this study is the large sample size of mumps-negative buccal swabs available for testing. Not all states have a laboratory available to perform mumps RT-PCR testing with the capacity to store the samples at a temperature in an environment to preserve them for further examination. Additionally, active surveillance for parotitis in the presence of the large mumps outbreak provided the opportunity for obtaining a more representative population-based sample than passive reporting of influenza-related parotitis cases would allow. Furthermore, demographic data collected during the course of the investigation, although planned for use in identifying mumps risk and not influenza, was a useful information source.

This study has several limitations. First, buccal swabs are not approved for influenza testing and might underestimate positive results; nasopharyngeal swabs or aspirates are preferred for influenza testing. More data is needed in the future regarding the sensitivity and specificity of buccal swabs for influenza virus detection. Second, funding constraints and a lack of available laboratory staff did not allow testing for other respiratory viruses or subtyping the identified influenza A viruses. Overall, Arkansas experienced a mild influenza season during 2016–−2017, and H3N2 was the most common subtype of influenza A identified in the state. Other studies have reported that Epstein Barr virus and parainfluenza viruses are most commonly isolated in cases of nonmumps parotitis, along with multiple other respiratory viruses and herpes viruses [2–6,8], and limitations in funding precluded us from testing more broadly for these other viruses. However, the attribution of causality to the presence of these viruses remains difficult to confirm, because there is evidence of asymptomatic shedding that complicates interpretation of test results [11]. Additionally, all available buccal swabs were tested, including those collected up to 21 days after onset of parotitis; these may represent false negatives for either mumps or influenza, as mumps virus shedding is known to decrease rapidly after onset of parotitis [13]. Finally, influenza vaccination status was not available to examine potential protective effects; a 2017 study of influenza parotitis showed no significant difference between patients who had received influenza vaccine versus matched controls without parotitis who had also received the vaccine [11]. Further study investigating the effects of influenza vaccination on influenza-related parotitisis warranted.

These data add to the growing body of literature demonstrating the burden of parotitis associated with influenza A. Although mumps remains the most commonly identified cause of parotitis in the United States and requires public health intervention, health care providers should consider testing for influenza in cases of sporadic parotitis.

## Figures and Tables

**Table 1:** Comparison of Demographic Characteristics of Influenza-Related Parotitis and Mumps Parotitis Cases — Arkansas, August 2016–July 2017.

	Male Sex No. (%)		Race No. (%)		Age in years median (range)	
**Mumps Parotitis** (N = 1665 buccal swab PCR positive for mumps)	883 (53)		323 (19) white 1036 (62) Native Hawaiian/Pacific Islander		15 (0–75)	
**Mumps negative and influenza negative parotitis** (N = 196) No. (%)	70 (36)	P = 0.04^1^	149 (76) white 14 (7) Native Hawaiian/ Pacific Islander	P = 0.61^1^	14 (0–90)	P<0.001^2^
**Influenza-Related Parotitis (N = 19 buccal swab PCR positive)** **No. (%)**	12 (63)		17 (89) white 0 (0) Native Hawaiian/Pacific Islander		12 (1–25)	

1 Calculated using chi-square test

2 Calculated using Wilcoxon Rank Sum test.
